# Beneficial effects of a prebiotic-postbiotic supplement on digestive health and fecal microbiota in dogs and cats

**DOI:** 10.3389/fvets.2026.1797178

**Published:** 2026-03-09

**Authors:** Céline S. Nicolas, Fanny Lloret, Thomas Carton, Lou Beuvin, Christophe A. Rème

**Affiliations:** 1 MU Petfood Petcare, Virbac SA, Carros, France; 2 Department of Biometry, Biofortis SAS, Saint-Herblain, France

**Keywords:** diarrhea, digestive issues, Floragest, gastrointestinal disorders, microbiome, nutraceutical, pets, stool consistency

## Abstract

Gastrointestinal (GI) disorders are a frequent concern for pet owners and veterinarians. Dietary management of mild GI disorders is often essential in order to correct imbalances in the gut flora. In this context, “biotics,” including probiotics, prebiotics, and postbiotics, have received increasing attention for their potential to favorably modulate the gut microbiota and support digestive function. In this study, we investigated the effect of a nutritional supplement containing a specific blend of prebiotics (baobab fruit pulp and acacia gum) and postbiotics (inactivated *L. helveticus* and selected yeast fractions) on digestive signs exhibited by healthy dogs and cats with mild GI imbalances, such as soft stools, increased quantity of stools or flatulence. The supplement was tested over a 28-day period. Digestive signs were evaluated on 57 dogs and 57 cats and the microbiota analysis was conducted on samples from 35 dogs and 27 cats. Questionnaires completed by the pet owners at regular intervals were used to evaluate digestive signs, while changes in the intestinal microbiota were assessed by fecal sample analysis performed before and after supplementation. The supplement was accepted either alone or mixed with food by 94% of dogs and 91% of cats. In both cats and dogs, the supplementation significantly improved digestive health as of day 7 (*p* < 0.001). Animals starting the study with impaired stool consistency or high quantity of stools showed significant improvement by day 7. The stool odor also improved from the first week. The proportion of dogs and cats with flatulence was reduced by 45 and 63%, respectively, by day 28 (*p* < 0.001), and the proportion of pets vomiting was reduced by 51 and 54%, respectively (*p* < 0.001). The impact on gut microbiota involved mainly changes on multiple subdominant taxa (such as *Megamonas* and *Peptacetobacter* in dogs, *Anaerotignum* and *Succinivibrio* in cats), without altering the overall microbial community architecture (as shown by diversity indices). Altogether, these results show that the supplement tested (Floragest™ soft chews, Virbac) can help support the digestive health of dogs and cats with mild gastrointestinal disorders.

## 1 Introduction

Companion animals, particularly dogs and cats, hold an important place in European households, with nearly half of families owning at least one pet (1). Their status within the home has continuously evolved, shifting from a purely utilitarian role to that of a full-fledged family member, and owners increasingly prioritize the health and wellbeing of their animals. Among the various aspects of pet health, gastrointestinal (GI) function represents a major concern for both owners and veterinarians.

GI disturbances are frequently reported in veterinary clinical practice. Analyses of primary care medical records in the United Kingdom indicate that enteropathies are among the most common disorders in dogs (prevalence of about 10%) (2), with the annual incidence of acute diarrhea estimated at approximately 8% (3). Clinical observations further suggest that vomiting and diarrhea together may account for up to 20–30% of veterinary consultations (3). Similar trends have been described in cats, for which GI signs also constitute a substantial proportion of routine veterinary presentations (prevalence of about 10% for mild-enteropathic disorders, 3% for diarrhea) (4, 5). Enteropathies are the third and fourth cause of consultation in primary care (2, 4).

Dietary approaches are an important strategy of GI management in dogs and cats (6). While full dietary modification or pharmacologic therapy is typically reserved for moderate to severe gastrointestinal disease (7), milder disturbances, such as intermittent soft stools or minor fluctuations of stool quality, are often managed through targeted nutritional supplementation (8–10). In this context, “biotics,” including probiotics, prebiotics, synbiotics and postbiotics, have received increasing attention for their potential to modulate the gut microbiota and support digestive function (11).

The GI microbiota of dogs and cats is a complex and dynamic ecosystem dominated by Bacillota (formerly named Firmicutes), Pseudomonadota (formerly named Proteobacteria) and Bacteroidota (formerly named Bacteroidetes), the most abundant phylum, followed by Fusobacteriota and Actinomycetota (formerly named Actinobacteria) (12–16). Although their roles remain poorly defined in dogs and cats, these microbial communities are known to contribute to host health through multiple mechanisms, including fermentation of non-digestible substrates, production of short-chain fatty acids (SCFAs), reinforcement of epithelial barrier integrity, immune modulation, and protection against pathogens in human (11, 17–22). Disruptions in microbiota composition or metabolic activity, collectively described as dysbiosis, are well documented in both acute and chronic GI disorders in dogs and cats (23–26). Dysbiosis typically involves reduced microbial diversity, decreased abundance of saccharolytic taxa, increased proteolytic or pathobiont populations, and alterations in SCFA profiles, which can contribute to soft or diarrheic stools (12, 13, 27).

Prebiotics, defined by the International Scientific Association for Probiotics and Prebiotics (ISAPP) as substrates selectively utilized by host microorganisms to confer a health benefit, are known to enhance saccharolytic fermentation, improve stool quality, and promote the growth of beneficial bacteria (28, 29). Their efficacy has been demonstrated in both dogs and cats, including reductions in pathogenic bacteria and increases in beneficial taxa (30–33).

Postbiotics, defined by the ISAPP as non-viable microorganisms, microbial components, or metabolites that confer a health benefit, also show promising effects in companion animals (34). In cats, a heat-killed *Bifidobacterium animalis subsp. Lactis* preparation has been reported to enhance gut barrier integrity, enrich beneficial bacteria like *Bifidobacterium* spp., and increase fecal concentrations of key SCFAs (35). In dogs, combinations of prebiotics and postbiotics shaped microbiota, and resulted in increased relative abundance of presumed beneficial microbes (36). A recent meta-analysis highlighted multiple benefits of postbiotic supplementation in dogs, particularly regarding modulation of the fecal microbiota, although no significant changes were observed in fecal consistency (37).

Despite these emerging findings, evidence supporting combined clinical and microbiome effects of prebiotic–postbiotic supplementation remains limited. Few studies have simultaneously assessed stool quality and microbiome composition in real-world conditions, and even fewer have included both dogs and cats.

The present study aimed to address this gap by evaluating the effects of a nutritional supplement containing a specific blend of prebiotics and postbiotics on digestive signs in pet dogs and cats with mild GI disturbances.

The test product, composed of baobab fruit pulp, acacia gum (gum arabic), inactivated *Lactobacillus helveticus* HA-122, and selected fractions of inactivated yeast strains, has previously been investigated *in vitro*, in the Simulator of the Canine Intestinal Microbial Ecosystem (SCIME®). It was shown to positively influence the activity and composition of the gut microbiome from canine donors with soft stools (38). In addition, repeated administration of baobab fiber and acacia gum significantly modulated microbial metabolic activity, diversity indices, and community structure in an experimental model, called Simulator of the Human Intestinal Microbial Ecosystem (SHIME®), notably enhancing *Bifidobacteriaceae* and *Faecalibacterium prausnitzii* levels (39). Both prebiotics seemed to have effects in different regions of the digestive tract model. Furthermore, both the probiotic live yeast *Saccharomyces boulardii* CNCM I-1079 and the postbiotic heat-inactivated *Lactobacillus helveticus* HA-122 were shown to accelerate the recovery of total bacterial load and the restoration of bacterial diversity in a canine-specific *in vitro* model following antibiotic administration (40).

From this basis, this study aimed at evaluating the effects of the test product *in vivo* in both healthy dogs and cats, on digestive health and fecal microbiota composition. Specifically, we sought to correlate changes in clinical parameters, such as stool quality, with shifts in fecal microbiota composition. This integrated approach is intended to provide new insights into the relationship between targeted nutritional supplementation, microbiota modulation, and digestive wellbeing in companion animals.

## 2 Materials and methods

### 2.1 Animals

The study included 64 owned dogs and 67 owned cats, older than 1 year of age, living in France. Dogs and cats could be of any breed and sex. For dogs, the three types of size had to be present in equal manner: small (<15 kg), medium (15–35 kg) and large (>35 kg); proportions are presented in Table 1. The pet owners participating in the study were recruited by an independent institute which also gathered all the data (Techni’sens, Bordeaux, France). Owners were included based on a pre-study questionnaire. Pets had to be overall healthy according to their owners, regularly wormed and not taking any treatment or specific diet, according to their owners. All animals were kept on their usual diet throughout the study; no dietary changes were allowed. To be included, the dogs and cats had to present at least one of the following signs: frequency of defecation ≥ three times a day; most frequent fecal score observed (based on the Waltham fecal scoring grid^1^) > 3; and/or presence of flatulence several times a week or every day. Since feces had to be observed regularly and fecal samples collected during the study, pets living mainly outdoors were excluded. Other exclusion criteria included pregnancy, lactation, allergies and daily vomiting. Only owners able to observe, smell and score their animals’ feces and flatulence were selected.

**Table 1:** Dogs and cats characteristics at baseline.

Characteristics		Dogs (*n* = 57)	Cats (*n* = 57)
Sex	Male	60%	56%
	Female	40%	44%
Sterilized	Yes	40%	89%
	No	60%	11%
Age (years)	1–5	47%	68%
	6–10	37%	18%
	>10	16%	14%
Body weight (kg)	Mean (SD)	25.1 (14.9)	5.1 (2.0)
Size	Small (<15 kg)	33%	/
	Medium (15–35 kg)	33%	/
	Large (>35 kg)	34%	/

### 2.2 Product tested

The test product, a feed supplement, was a soft chew containing a proprietary mixture with baobab fruit pulp and acacia gum (or gum arabic) as prebiotics, and heat-killed whole cell *L. helveticus* HA-122, and specific fractions of the three inactivated yeast strains *S. cerevisiae* AQP 12260, *S. cerevisiae* AQP 12988, and *C. jadinii* AQP 12549, as postbiotics. The test product (Floragest™ soft chews) was provided by Virbac (Carros, France) in white unlabeled jars so that the participants were blinded to the name, composition of the product, and supplier. The supplement was provided in different sizes: 0.9 g chews for cats, 2 g chews for small dogs (<15 kg), 4 g chews for medium dogs (15–35 kg) and 6 g chews for large dogs (>35 kg). Every owner was instructed to give one soft chew per day to their pet.

### 2.3 Assessments

At the beginning of the study (D0), and after 7, 14 and 28 days, owners were asked to fill in a questionnaire assessing different aspects of their pet’s digestive health. They were asked to score the following: the overall digestive health of their pets using an 11-point scale (0—very bad to 10—very good); the daily stool quantity using a 5-point scale (1—very low to 5—very high); the stool consistency using a 9-point scale (1—very dry, hard and crumbly stools to 5—completely liquid stools), based on the Waltham fecal scoring system (see text footnote 1), using half-point increments; and the stool odor using a 5-point scale (1—very low odor, very acceptable to 5—very malodorous, unbearable). They also reported pet’s flatulence and episodes of vomiting, if any. Owners were asked to report bad breath odor frequency at D0 (every day, several times per week, occasionally, or never) and to assess changes at D28 (markedly improved, slightly improved, no change, slightly worsened, or markedly worsened). Finally, the acceptability of the supplement and overall satisfaction with the product was rated.

On D0 and D28, owners collected a stool sample to be sent for analysis. For cats, they were instructed to place paper on the litter beforehand, and to only collect feces which were not in direct contact with the litter. Briefly, fecal samples were collected using a 1 g calibrated spoon and immediately transferred into 1 mL of preservation reagent (DNA/RNA Shield). Samples were maintained at room temperature (15–25 °C) and shipped to the laboratory for analysis. DNA was extracted using the ZymoBIOMICS 96 MagBead DNA Kit (Zymo Research Corp., USA). DNA isolation was carried out on a KingFisher Flex automated station (ThermoFisher Scientific Inc., USA) according to the manufacturer’s instructions. The V3–V4 regions of the gene encoding 16S ribosomal RNA was amplified by polymerase chain reaction (PCR) using primers 341F and 785R (41) and sequenced on Illumina MiSeq (Illumina, USA). The targeted sequences from microbiota were analyzed using a bioinformatic pipeline developed by Biofortis based on Dadaist2 software (42). Further details are provided in Supplementary material.

### 2.4 Statistical analysis

Questionnaires: Due to the nature of the data (ordinal or nominal) and non-normal distribution of the data (verified with the Shapiro–Wilk test), non-parametric tests were performed. A Friedman test was used to assess the evolution of scores (digestive health score, stool odor score). In case of significance, Wilcoxon Signed-Rank Tests were used for pairwise comparisons of each subsequent time-point to baseline (D0). *p*-values from these comparisons were adjusted using the Benjamini-Hochberg procedure to control the false discovery rate. Cochran’s *Q* test was used to assess the changes in frequencies of signs (pets with moist or dry stools, high or low quantity of stools, or with episodes of vomiting, flatulence or bad breath). If significant, McNemar’s tests were then used for pairwise comparisons with the Benjamini-Hochberg procedure to adjust *p*-values. Data are presented as median (first quartile Q1–third quartile Q3) or as percentage of respondents reporting the sign. The data analysis for this part was generated using the Real Statistics Resource Pack software (Release 8.9.1; Charles Zaiontz).

Microbiota analysis: The α-diversity indices used in this analysis were Observed, Inverse Simpson, Shannon, and Phylogenetic Diversity (PD) (43) [see Magurran (44) for a general overview]. Changes of α-diversity from baseline were evaluated using a nonparametric Wilcoxon test. Only subjects with valid data on both visits were included in the analysis. The dissimilarity measures (β-diversity indices) used in this analysis were Bray-Curtis, Jaccard and Weighted UniFrac (45) [see Legendre and Legendre (46) for a general overview]. For each individual, β-diversity indices were used to quantify the changes in gut microbiota composition from D0 to D28. Significance of those changes was then evaluated using a non-parametric Wilcoxon test. Statistical model for comparing changes in abundance is described in Supplementary material. Only taxa present in at least 30% of the samples were included in the analysis.

## 3 Results

### 3.1 Animals’ characteristics

Over the 64 dogs and 67 cats included initially, 57 dogs and 57 cats completed the study. Two dogs and six cats refused to take the supplement more than 50% of the time, three dogs and two cats showed signs of intolerance, one dog had to be hospitalized for reasons unrelated to the chew, and one dog and two cats died due to circumstances unrelated to the product or were lost during the study. These pets were withdrawn from the study and were therefore removed from the efficacy analysis (but kept for the acceptability data on D0).

**Figure 1:**
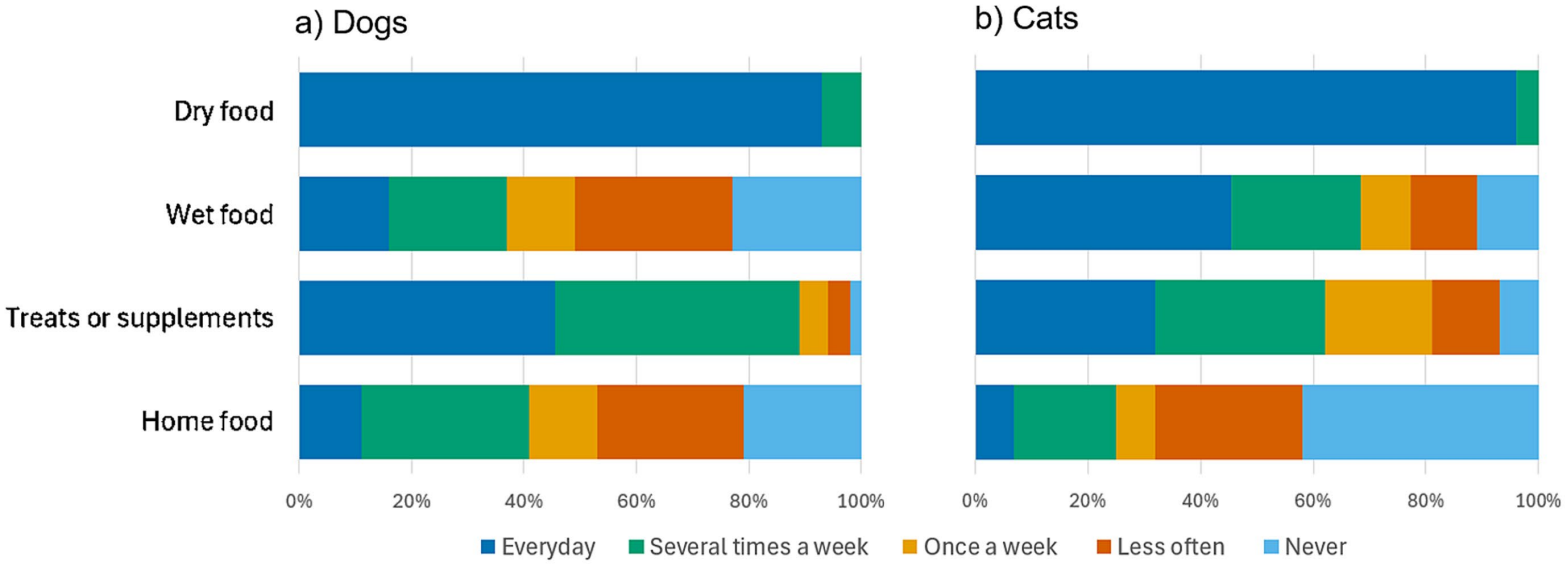
Consumption frequency of different food types in dogs and cats: (a) Dogs, *n* = 57; (b) Cats, *n* = 57.

The pets’ characteristics and type of food they usually received are presented in Table 1 and Figure 1. All of them were fed with dry food every day or several times a week and most of them also regularly received wet food (especially cats), treats or supplements (especially dogs).

At baseline, among the 35 dogs with exploitable microbiota results, 26% had moist or liquid stools, 3% had hard stools, 29% had important quantity of stools, 45% had stools with a strong unpleasant odor, 97% experienced flatulence, and 69% had episodes of vomiting. Among the 27 cats, 15% had moist or liquid stools, 26% had hard stools, 19% had important quantity of stools, 44% had stools with a strong unpleasant odor, 78% experienced flatulence, and 70% had episodes of vomiting.

### 3.2 Acceptability of the supplement

From original data set (64 dogs and 67 cats), on D0, 94% dogs and 91% cats accepted the supplement either alone (84.5% of dogs and 75% of cats) or mixed with food (9.5% of dogs and 16% of cats). Considering only animals who finished the study (57 dogs and 57 cats), on D0, 96.5% of dogs accepted the supplement either alone (87.5%) or mixed with food (9%) while by D28, they all accepted the supplement (89% alone and 11% with food). For cats, on D0, 95% accepted the supplement either alone (77%) or mixed with food (18%) while by D28, 98% accepted the supplement (86% alone and 12% with food). There was a positive evolution of the chew acceptability over time, observed by 46% of dog owners and 56% of cat owners. The median (Q1–Q3) acceptability score given by owners was 9 (8–10)/10 for both dogs and cats, and 93% of dog owners and 82% of cat owners thought their pet took the product easily.

### 3.3 Digestive health and signs

For both cats and dogs, the median (Q1–Q3) score given for digestive health increased from 7 (6–8) to 8 (7–9)/10 between D0 and D28, with a significant improvement over time (*p* < 0.0001) observed as early as D7 (see Tables 2, 3). By D28, 65% of dog owners and 58% of cat owners noted an improvement in their pet’s digestive health.

**Table 2:** Scores and signs reported by dog owners.

Signs reported (dogs)	Day 0	Day 7	Day 14	Day 28	*p*-value (a)
Digestive health—score/10	7 (6–8)	8 (7–8)***	8 (7–9)***	8 (7–9)***	<0.0001
Pets with very moist or liquid stools (score >3)	23%	19%	18%	12%	0.35
Pets with dry, hard stools (score <2)	2%	9%	7%	18%*	0.0095
Pets with an ideal score (2–3) among those impaired at the beginning (score <2 or >3) (*n* = 14)	0%	43%*	64%*	64%*	0.0015
Pets with high quantity of stools	25%	19%	16%	18%	0.45
Pets with normal or low quantity of stools while they were high at the beginning (*n* = 14)	0%	43%*	64%*	64%*	0.0007
Stool odor—score/5	3 (3–4)	3 (2–3)***	3 (2–3)***	3 (2–3)***	<0.0001
Pets having stools with a strong, unpleasant odor	37%	16%**	11%**	11%**	<0.0001
Pets with flatulence	96%	37%***	53%***	51%***	<0.0001
Pets vomiting	67%	5%***	11%***	16%***	<0.0001

Data are expressed as median (Q1–Q3) scores or as the percentage of pets showing the sign, according to their owner (*n* = 57 unless otherwise stated). (a) *p*-value obtained with Friedman’s or Cochran’s *Q* test. *; **; ***: *p* < 0.05; 0.01; or 0.001, respectively, compared to Day 0 (adjusted p-values from Wilcoxon signed-rank test or McNemar’s test).

**Table 3:** Scores and signs reported by cat owners.

Signs reported (cats)	Day 0	Day 7	Day 14	Day 28	*p*-value (a)
Digestive health—score/10	7 (6–8)	8 (7–8)**	8 (7–9)***	8 (7–9)***	<0.0001
Pets with very moist or liquid stools (score >3)	18%	12%	9%	9%	0.33
Pets with dry, hard stools (score <2)	28%	21%	16%	21%	0.19
Pets with an ideal score (2–3) among those impaired at the beginning (score <2 or >3) (*n* = 26)	0%	54%***	58%***	62%***	<0.0001
Pets with high quantity of stools	18%	5%	7%	7%	0.049
Pets with normal or low quantity of stools while it was high at the beginning (*n* = 10)	0%	100%**	100%**	90%**	<0.0001
Stool odor—score/5	3 (3–4)	3 (2–3)***	3 (2–3)***	2 (2–3)***	<0.0001
Pets having stools with a strong, unpleasant odor	32%	16%*	7%**	12%*	0.0001
Pets with flatulence	75%	12%***	14%***	12%***	<0.0001
Pets vomiting	65%	14%***	16%***	11%***	<0.0001

Data are expressed as median (Q1–Q3) scores or as the percentage of pets showing the sign, according to their owner (*n* = 57 unless otherwise stated). (a) *p*-value obtained with Friedman’s or Cochran’s *Q* test. *; **; ***: *p* < 0.05; 0.01; or 0.001, respectively, compared to Day 0 (adjusted p-values from Wilcoxon signed-rank test or McNemar’s test).

The proportion of dogs with very moist or liquid stools (fecal score >3) decreased from 23 to 12% between the beginning and end of the study. In cats, this ratio fell from 18 to 9%. The proportion of dogs with dry or hard stools (score <2) significantly increased over time (*p* = 0.0095) and on D28 (*p* = 0.0476), and 47% of dog owners thought the stools were firmer by D28. Conversely, the proportion of cats with dry or hard stools (score <2) did not change significantly over time but 37% of cat owners estimated that the stools were more consistent. Among the 14 dogs and 26 cats that started with a fecal score <2 or >3, 9 dogs (64%) and 16 cats (62%) ended up within the range 2–3 considered optimal (*p* = 0.0015). The increase became significant as early as D7 (Tables 2, 3).

Stools quantity did not significantly change over time for dogs. The percentage of cats with high quantity of stools significantly decreased over time (*p* = 0.049) but pairwise comparisons versus D0 did not show any significant difference. However, among the 14 dogs (25%) and 10 cats (18%) who had high quantity of stools at D0, 9 dogs (64%) and 9 cats (90%) were found to have normal or low quantity of stools by D28 (*p* < 0.001). This improvement was already significant by D7 (Tables 2, 3).

The score given to the stools odor significantly improved over time (*p* < 0.001), and as early as D7, in both dogs and cats (Tables 2, 3). At the beginning of the study, 37% of dog owners and 32% of cat owners considered the stools to have a strong and unpleasant odor. By D28, these ratios went down to 11 and 12%, respectively (*p* < 0.001).

The proportion of owners reporting their dog had flatulence decreased from 96 to 51% and from 75 to 12% in cats (*p* < 0.0001 in both species). The reduction in flatulence was significant as of D7 in both species. Similarly, the percentage of owners reporting vomiting went from 67 to 16% in dogs and from 65 to 11% in cats (*p* < 0.0001 in both species), with a significant improvement as of D7. Regarding breath odor, at D0, 28% of owners considered their dogs to have bad breath every day, 21% several times per week, 37% occasionally and 14% never. Among cats owners, 11% reported daily bad breath, 16% several times per week, 37% occasionally and 26% never. By D28, an improvement in breath odor was reported by 54 and 40% of dogs and cats owners, respectively.

### 3.4 Satisfaction

The median (Q1–Q3) satisfaction score given by owners was 8 (7–9)/10 for dogs and 8 (8–9)/10 for cats. After 1 month, 89 and 88% of dog and cat owners would recommend the product, respectively, and 93 and 86% would buy it if recommended by a veterinarian.

### 3.5 Microbiota analysis

#### 3.5.1 Dogs

Among the 57 dogs that completed the study, fecal samples were available at both timepoints for 35 animals.

Analysis of dogs’ fecal microbiota profiles revealed minor changes in microbial diversity and taxonomic composition over the 28-day supplementation period.

Measures of α-diversity (Observed richness, Shannon, and PD) showed overall stable diversity profiles between D0 and D28. None of the indices demonstrated statistically significant differences after correction for multiple testing, indicating that the dominant structure of the microbiota remained globally stable throughout the study period (Supplementary Figure 1).

Global comparisons of microbiota composition using Bray–Curtis and Jaccard distances (β-diversity indices) confirmed the overall stability of the community structure across timepoints (Supplementary Table 1). While interindividual variability remained high, no consistent directional shift emerged in the cohort, suggesting that supplementation did not markedly alter the overall microbial community architecture.

Regarding taxonomic composition, statistically significant differences were observed between day 0 and day 28 after correction for multiple testing (Table 4). The relative abundance of *Megamonas*, particularly *M. fusiformis*, increased from 6.7 to 9.6% (adjusted *p* = 0.0108). We also observed a slight increase in the relative abundance of *Peptacetobacter*, particularly *P. hiranonis* and *Kineothrix*, particularly *K. alysoides* (adjusted *p* = 0.0299 for both). Additionally, while the change did not remain significant after correction for multiple comparison, the relative abundance of *Clostridium sensu stricto* notably decreased from approximately 4% to less than 1% (adjusted *p* = 0.0761).

**Table 4:** Relative abundance changes from baseline of fecal microbiota genera in dogs.

Taxon	Direction	Mean abundance		Prevalence		Model effect size	*p*-value	Adjusted *p*-value
		D0	D28	D0	D28			
Megamonas	↑	6.66	9.59	80.00	100.00	1.10	2e-04	**0.0108**
Peptacetobacter	↑	7.14	7.67	91.43	100.00	0.48	0.0015	**0.0299**
Laedolimicola	↓	0.02	0.01	37.14	31.43	−0.66	0.0025	**0.0299**
Kineothrix	↑	0.64	0.96	82.86	94.29	0.67	0.0025	**0.0299**
Terrisporobacter	↓	0.24	0.09	48.57	20.00	−0.90	0.004	**0.0376**
Enterocloster	↓	0.02	0.01	25.71	25.71	−0.44	0.0049	**0.0383**
Thomasclavelia	↑	0.43	0.47	71.43	88.57	0.73	0.0089	0.0599
Clostridium sensu stricto	↓	3.99	0.78	88.57	85.71	−1.00	0.0129	0.0761
Vescimonas	↓	0.03	0.02	31.43	31.43	−0.50	0.0154	0.0803
Intestinimonas	↓	0.01	0.01	40.00	42.86	−0.54	0.0207	0.0971
Allobaculum	↓	0.79	0.55	57.14	68.57	0.75	0.0312	0.1332

The indicated direction of change in abundance corresponds to the average observed difference in relative abundance between visits. Mean abundance and prevalence are displayed as percentage (%). Only taxa with a prevalence of 20% or more are considered. In this table, only results with a *p*-value < 0.05 are shown. Bold values: significant difference after *p*-value adjustment.

#### 3.5.2 Cats

Fecal samples at both timepoints were available for 27 of the 57 cats that completed the study. Across the feline cohort, analysis of fecal microbiota profiles also revealed some subtle changes over the 28-day supplementation period.

Overall measures of α-diversity (Observed richness, Shannon, and inverse Simpson indices) remained largely stable between D0 and D28, indicating that the dominant microbial community structure was maintained (Supplementary Figure 2). Some trends were notable at the biological level: there was a slight decrease in the number of subdominant species, reflected by minor reductions in Observed richness, suggesting a minor loss of low-abundance taxa, while evenness remained largely unchanged (as shown by Shannon and Inverse Simpson indices).

Global comparisons of β-diversity indices (Jaccard and Bray–Curtis distances) confirmed the absence of major restructuring of the microbiota across timepoints (Supplementary Table 2). Interindividual variability was high, and changes were subtle, suggesting that supplementation did not induce pronounced shifts in community composition.

At the taxonomic level, only a limited number of significant or near-significant changes were observed between D0 and D28 (Table 5). The relative abundance of the genus *Anaerotignum*, particularly the species *A. lactatifermentans*, as well as that of the genus *Succinivibrio*, specifically *S. dextrinosolvens*, decreased after 28 days of supplementation (adjusted *p* = 0.0233 and 0.0557, respectively). Beyond strict statistical significance, several biologically relevant trends were identified. The dominant genus *Catenibacterium* showed an increase over the study period, rising from 3.3 to 5.0% (adjusted *p* = 0.1515). Conversely, a reduction in *Phascolarctobacterium*, particularly *P. succinatutens*, was also observed (adjusted *p* = 0.1072).

**Table 5:** Relative abundance changes from baseline of fecal microbiota genera in cats.

Taxon	Direction	Mean abundance		Prevalence		Model effect size	*p*-value	Adjusted *p*-value
		D0	D28	D0	D28			
Anaerotignum	↓	0.16	0.05	81.48	70.37	−0.81	3e-04	**0.0233**
Succinivibrio	↓	0.79	0.32	55.56	44.44	−1.01	0.0014	**0.0557**
Phascolarctobacterium	↓	1.89	1.41	77.78	55.56	−1.23	0.0048	0.1072
Allisonella	↓	0.14	0.08	85.19	62.96	−0.93	0.0054	0.1072
Ruthenibacterium	↑	0.02	0.11	37.04	62.96	0.68	0.0081	0.1275
Helicobacter	↑	0.14	0.22	62.96	62.96	0.79	0.0096	0.1275
Catenibacterium	↑	3.33	5.03	85.19	92.59	0.97	0.0133	0.1515
Hydrogeniiclostridium	↑	0.00	0.05	25.93	33.33	0.69	0.0176	0.1758
Mediterraneibacter	↓	0.40	0.24	96.30	100.00	−0.41	0.0286	0.2543
Solibaculum	↑	0.13	0.16	66.67	81.48	0.47	0.039	0.2872
Clostridium sensu stricto	↓	1.65	1.07	96.30	88.89	−0.88	0.0395	0.2872
Enterococcus	↑	0.11	0.23	55.56	62.96	0.87	0.0482	0.3211

The indicated direction of change in abundance corresponds to the average observed difference in relative abundance between visits. Mean abundance and prevalence are displayed as percentage (%). Only taxa with a prevalence of 20% or more are considered. In this table, only results with a *p*-value < 0.05 are shown. Bold values: significant difference after *p*-value adjustment.

These observations show, in both the microbiota of dogs and cats, a subtle modulation of low-abundance taxa, potentially reflecting targeted effects of the supplement on the microbiome, and not a large-scale community change.

## 4 Discussion

Dietary-based strategies are a valuable option to address mild GI disorders, a frequent reason for veterinary consultation in companion animals. Although probiotics have been extensively studied, evidence regarding the efficacy of prebiotics and postbiotics, alone or in combination, on GI symptoms and gut microbiota composition remains limited. This study aimed to begin addressing this gap by evaluating the effects of heat-killed *Lactobacillus helveticus* HA-122, and selected fractions of inactivated yeast strains (*Saccharomyces cerevisiae* AQP 12260 and AQP 12988, *Cyberlindnera jadinii* AQP 12549) on the digestive health of domestic dogs and cats when given with prebiotics (baobab fruit pulp and acacia gum).

Overall, 28 days of supplementation resulted in very significant GI improvements, as reported by about 70 out of 114 owners. The proportion of animals with moist or liquid stools was significantly reduced after the intervention period, in both dogs and cats. Nearly half as many pets had very moist or liquid stools after the supplementation period, as compared to baseline, while dry and hard stools became more frequent over time. Altogether, the data indicates that the intervention tended to normalize stool consistency. The impact of prebiotics on stool consistency was already observed in other studies. Administration of complex carbohydrates (phosphorylated mannanoligosaccharides) was associated with rapid remission in dogs with diarrhea induced by pathogenic bacteria (30). In another study performed on 32 senior dogs, a prebiotic fiber blend (sugar beet pulp, GOS, and cellulose) improved stool consistency after a 3-week supplementation (47). This effect was evidenced by a lower proportion of dogs with poor fecal scores (≤1.5 or ≥3.75 on the Waltham scale) in the supplemented group compared with the control group. Although our study was not controlled, it provides further promising results on the benefit of pre-/postbiotic combination to regulate stool consistency in dogs and/or cats.

In addition, we report a regulating impact of the dietary intervention on stool quantity, but also flatulence, smelly stools and vomiting. This has already been observed in one study on healthy dogs, where an 8-week supplementation with prebiotics (galacto-oligosaccharide) significantly improved fecal odor (48). Finally, animals’ breath odor also appeared to improve with supplementation. To our knowledge, no studies have specifically investigated the effects of prebiotics and/or postbiotics on bad breath in animals with moderate GI disorders. However, previous observations suggest that a two-week postbiotic supplementation (dried *Pediococcus pentosaceus* fermentation product, and dried *Bacillus subtilis* fermentation product) may reduce halitosis in dogs (49).

For most of the digestive evaluations performed during the study, owners were able to observe an improvement as early as 1 week after the start of supplementation, which is quite rapid. Although the uncontrolled design of our study limits the ability to draw causal conclusions, the improvements observed in GI symptoms are of interest and deserve further confirmative studies.

Regarding fecal microbiota composition, the 28-day supplementation in dogs was associated with minor modulation, characterized primarily by changes in specific low-abundance taxa rather than broad restructuring of the microbial community. First, we observed an increase in relative abundance of *Megamonas*, particularly *M. fusiformis*, a SCFA-producing species (50). The relative abundance of *P. hiranonis* (formerly *Clostridium hiranonis*) also increased over the study period: this can be considered as a positive impact. Indeed, *P. hiranonis* is the main, and possibly the only, bacterial species responsible for bile acid metabolism in domestic dogs and is recognized for its beneficial effects on canine gut health (51–53). Dogs with chronic enteropathies or antibiotic-induced dysbiosis exhibit a reduced relative abundance of *P. hiranonis*, which is associated with a decreased proportion of secondary bile acids in the colon (54, 55). Secondary bile acids could exert several beneficial effects, such as anti-inflammatory actions across various organ systems, inhibition of *Clostridium difficile*, *Clostridium perfringens*, and *Escherichia coli* growth, and modulation of pancreatic glucose and insulin secretion (56). *P. hiranonis* was found to be significantly increased following dietary treatment in dogs suffering from chronic enteropathy (57). In addition, we also observed a significant increase in *Kineothrix alysoides* relative abundance, a species found to be more abundant in healthy dogs than in dogs with lymphoma (58). This species has been shown to mitigate liver dysfunction in mice suffering from liver disease (59).

In cats, the supplementation was also associated with selective modulation of specific low-abundance taxa. The relative abundance of *Anaerotignum* genus significantly decreased, driven by the species *A. lactatifermentens*, whose abundance was reported to increase in pathological contexts in humans (60) and turkeys (61). Moreover, the relative abundance of *Succinivibrio dextrinosolvens*, a species recently found to be overrepresented in cats with lithiasis, compared to healthy cats, slightly decreased over the supplementation period (62). Beyond strict statistical significance, some biologically relevant findings have emerged. We notably observed an increase in the relative abundance of *Catenibacterium*, a genus associated with a healthy feline gut microbiota (63). This genus contains only one species, known to be SCFA producer (64).

These findings indicate a limited influence of the supplementation on canine and feline gut microbial balance, without major changes in overall diversity or dominant microbial composition, when given to healthy pets with minor digestive issues. Indeed, no significant modulations were observed, either on α or β-diversity indices. However, the fine and selective modulation of some specific species may support the observed health benefits. The biological mechanism involved in the relationship between microbiota changes and clinical improvement remains to be elucidated.

Digestibility, which is influenced by the physicochemical properties and composition of both the supplement and the animal’s habitual diet, may represent an important modulating factor of the product’s effect on digestive outcomes and gut microbiota composition. Thermal and mechanical processing methods such as baking or extrusion are known to enhance carbohydrate digestibility by disrupting complex structures and increasing starch availability (65, 66). In contrast, when applied to certain carbohydrate sources, these processes may reduce protein digestibility, thereby altering the overall balance of nutrient utilization (67). The limited contribution of the supplement to the overall diet, combined with the lack of a standardized ration, likely resulted in marked inter-individual differences in digestibility. This heterogeneity may partly account for the inter-individual variability observed in response to supplementation. In light of these results, studies using more controlled nutritional conditions, particularly a standardized diet, may provide additional insight and allow a more refined evaluation of the product’s effects.

Our study has several other limitations that should be acknowledged. First, the relatively small number of animals included in the study, especially for microbiota outcomes, may have reduced the statistical power, making it difficult to detect subtle changes or variations. Indeed, detecting modest effects often requires substantially larger populations (68). Second, only a small proportion of animals had moist or liquid stools on D0, suggesting that their gut function was largely normal and, by extension, that their microbiota was probably stable and eubiotic, reducing the potential for noticeable improvement. Indeed, among the 35 dogs and 27 cats whose microbiota could be analyzed, a minority had moist stools (≤26%) or a high defecation frequency (≤29%).

The minor changes observed in the microbiota structure may be partly explained by substantial inter-individual variability in gut microbiota and host response. Such variability, which is well described in humans but less so in companion animals, can attenuate detectable effects, particularly in heterogeneous study samples (69, 70). We can hypothesize that the observed average effects were driven by certain animals, while others showed little or no response. Indeed, it is well recognized that baseline microbiota composition can influence the response to supplementation (71, 72). This inter-individual variability may be further exacerbated by the relatively small sample size.

Overall, the modest effects observed are consistent with the nature of the intervention, which is not expected to induce changes of the magnitude seen with more intensive approaches, such as fecal microbiota transplantation or antibiotic treatment (73, 74).

Although the overall microbial structure remained largely stable, subtle functional or compositional changes at lower taxonomic levels cannot be ruled out and should be explored in future studies.

## 5 Conclusion

The product was very well accepted, with 94% of dogs and 91% of cats consuming the chew either alone or mixed with food on the first day. According to owner assessments, the product improved digestive health. Animals with moist stools or with high quantity of stools improved with the supplement. In addition, owners reported diminished stool odors, along with a marked reduction in flatulence and vomiting in both dogs and cats. Altogether, these results indicate that the 28-day supplementation had a great impact on digestive signs in dogs and cats with mild GI disturbances. The effect of the product on the gut microbiota appeared limited; however, some changes were observed, particularly among less abundant genera. Although these findings are difficult to interpret considering the limitations of the study and variability between individuals, the product showed potential beneficial effects on the microbiota and significant effects on digestive signs that warrant further investigation in a larger-scale study.

## Data Availability

The raw data supporting the conclusions of this article will be made available by the authors without undue reservation.

## Footnotes

1
https://www.waltham.com/s3media/2020-05/waltham-scoring.pdf
