# Supplementary material for: Beneficial effects of a prebiotic-postbiotic supplement on digestive health and fecal microbiota in dogs and cats
Source: Front Vet Sci. 2026 Mar 9;13:1797178. doi: 10.3389/fvets.2026.1797178 (PMC13006246; doi:10.3389/fvets.2026.1797178)
Supplement: Supplementary file 1 [file Data_Sheet_1.docx]

Supplementary Material

# Supplementary Data

**Material and methods**

**DNA extraction:** DNA was extracted using the ZymoBIOMICS™ 96 MagBead DNA Kit (Zymo Research Corp., USA) following a protocol with dual cell lysis (mechanical and chemical) using a FastPrep homogenizer (MP Biomedicals, USA). DNA isolation was carried out on a KingFisher Flex automated station (ThermoFisher Scientific Inc., USA) according to the manufacturer’s instructions. DNA was quantified by fluorimetry using a Qubit 2.0 (Thermo Fisher Scientific, USA).

**Library preparation and sequencing:** The V3-V4 regions of the gene encoding 16S ribosomal RNA was amplified by polymerase chain reaction (PCR) using primers 341F and 785R in a 25 µL reaction volume containing 12.5 ng of sample DNA (Klindworth et al. 2013). PCR cycling was performed with a first denaturation step at 95 °C for 5 minutes, followed by 25 cycles at 98 °C for 30 seconds, annealing at 55 °C for 30 seconds, elongation at 72 °C for 30 seconds, and a final extension of 72 °C for 5 minutes. The 16S rDNA gene amplicon library was generated for each sample by adding dual indices and Illumina sequencing adapters using the Nextera XT Index kit. Each library was cleaned up with magnetic AMPure XP beads (Beckman Coulter, France) and the size was verified by capillary electrophoresis with a 2100 Bioanalyzer (Agilent Technologies, USA). After quantification by fluorimetry (Qubit® 2.0 Fluorometer), libraries were normalized to 4 nM and pooled before denaturing and sequencing (2 x 250 paired-end, v2 chemistry) using an Illumina MiSeq (Illumina, USA).

**Data processing:** The targeted sequences from microbiota were analyzed using a bioinformatic pipeline developed by Biofortis based on Dadaist2 software (Ansorge et al. 2021). Basically, after demultiplexing the barcoded Illumina paired reads, single read sequences were paired for each sample into longer fragments and cleaned. After quality-filtering and sequencing error modeling, amplicons variants sequences (ASV) were obtained. A taxonomic assignment of these ASV was performed in order to determine bacterial community profiles.

Tools for bioinformatic pipeline and corresponding parameters are detailed below:

- FastQC v0.11.8: quality control report of NGS sequencing data.
- Dadaist2 Software version: 1.1.0.
- Database version used for taxonomy: RDP release 19.

**Statistical tests**

α-diversity analysis

The following α-diversity indices are considered (see Magurran, 2004. for a general overview (3)):

- **Observed**: The Observed index is an estimator of richness given by the number of components observed in each sample (number of ASVs). For an abundance profile $p=\left( p_{1},\ldots,p_{S} \right),p_{i}>0$, it is determined as:

$$O\left( p \right)=S.$$

The greater $O\left( p \right)$, the greater the richness.

- **Inverse Simpson**: This diversity index can be understood as the number of dominant components in terms of evenness. It is computed as

$$2D\left( p \right)=\frac{1}{\sum_{i=1}^{S} p_{i}^{2}},$$

where $\left( p_{i} \right)$ is the relative abundance of the $i$-th component and $S$ is the total number of components in the sample. In an ecosystem with a perfect equitability, the $2D$ index will be equal to the *Observed richness* ($O\left( p \right)$) index.

- **Shannon**: The Shannon index is a diversity estimator that incorporates both the richness and the equitability of the components present in each sample. It is calculated using the following formula:

$$H\left( p \right)=-\sum_{i=1}^{S} p_{i}{log}_{b}p_{i},$$

Here, $p_{i}={n_{i}}/N$ is the estimated proportion of components, $n_{i}$ is the number of reads in a component and $N$ is the total number of reads. $S$ is the number of components so that $\sum_{i=1}^{S} p_{i}=1$, and $b$ is the base of the logarithm. Here, we apply the natural logarithm, ${log}_{e}$.

The higher the value of the index, the greater the diversity.

- Phylogenetic diversity (PD) (see Faith, 1992 (4)): it represents the sum of the lengths of all branches on the phylogenetic tree. It is defined as:

$$H\left( p \right)\mathcal{=-\sum l}\left( b \right)\cdot p\left( b \right)\cdot lnp\left( b \right),$$

where $T$ is a rooted phylogenetic tree for the community, $\mathcal{l}\left( b \right)$ is the length of a branch $b$ of $T$, and $p\left( b \right)$ proportion of individuals in the community who are represented by leaves descending from $b$.

Changes of α-diversity from baseline were evaluated using a nonparametric Wilcoxon test. Only subjects with valid data at both visits were included in the analysis.

β-diversity analysis

β-diversity indices explore inter-individual (or inter-sample) diversity and they express, in particular, their dissimilarity in terms of richness, evenness or phylogeny. The dissimilarity measures used in this analysis are defined as follows for every pair of abundance profiles (**n***^A^,***n***^B^*) (see Legendre and Legendre, 2012 for a general overview (5)):

- **Bray-Curtis:** it is an abundance driven measure defined as

$$d_{BC}\left( A,B \right)=\sum_{j=1}^{S} w_{j}\frac{\left| n_{j}^{A}-n_{j}^{B} \right)}{\left( n_{j}^{A}+n_{j}^{B} \right)},$$

where $n_{j}^{A}$ is the number of reads for taxon $j$ in sample $A$ and

$$w_{j}=\frac{\left( n_{j}^{A}+n_{j}^{B} \right)}{\sum_{k=1}^{S} \left( n_{k}^{A}+n_{k}^{B} \right)}.$$

The greater the Bray-Curtis index, the further the samples/individuals are from each other in terms of richness and/or evenness.

- **Jaccard**: it is a presence/absence measure defined by

$$d_{J}\left( A,B \right)=\frac{a+b}{a+b+c},$$

where $a$ is the number of species that are present only in $A$, $b$ the number of those only in $B$ and $c$ the number of species found in both samples. The greater the Jaccard distance, the further the samples/individuals are from each other in terms of richness. Indeed, two samples/individuals containing exactly the same taxa will have a distance equal to $0$; conversely, two samples/individuals not sharing any species will have a distance equal to $1$.

- **Weighted UniFrac** (see Lozupone et al., 2007 (6)): this index takes into account presence, abundance and phylogenetic information to assess the dissimilarity between two taxonomic profiles. It is given by:

$$d\left( A,B \right)=\sum_{j=1}^{S} w_{j}\left( \nu\right)\frac{\left| q_{j}^{A}-q_{j}^{B} \right)}{\left( q_{j}^{A}+q_{j}^{B} \right)},$$

with

$$w_{j}=\frac{\mathcal{l}\left( j \right)\left( q_{j}^{A}+q_{j}^{B} \right)}{\sum_{k=1}^{S} \left( q_{k}^{A}+q_{k}^{B} \right)}.$$

In these expressions, $\mathcal{l}\left( j \right)$ is the length of the $j$-th branch of the phylogenetic tree and $q_{j}^{A},q_{j}^{B}$ are the branch proportions for community A and B, respectively. The index takes values between $0$ (no dissimilarity) and $1$ (maximum dissimilarity).

Tests performed on β-diversity:

**Comparison of changes from baseline**: for each individual, β-diversity indices are used to quantify the changes in their gut microbiota composition from visit D0 to D28. Significance of those changes is then evaluated using a non-parametric Wilcoxon test.

Abundance analysis

Statistical model for comparing changes in abundance is:

$$\Delta Y_{D28-D0}=Y_{D0}+\varepsilon_{i},$$

where $\Delta Y_{D28-D0}$ indicates the change in CLR-transformed relative abundances from D0 to D28, $Y_{D0}$ is the CLR-transformed relative abundance of the component at baseline and $\varepsilon_{i}$ is a zero-mean random term to account for the variability around the mean. Only taxa present in at least 30% of the samples are included in the analysis.

# Supplementary Figures and Tables

## Supplementary Figures


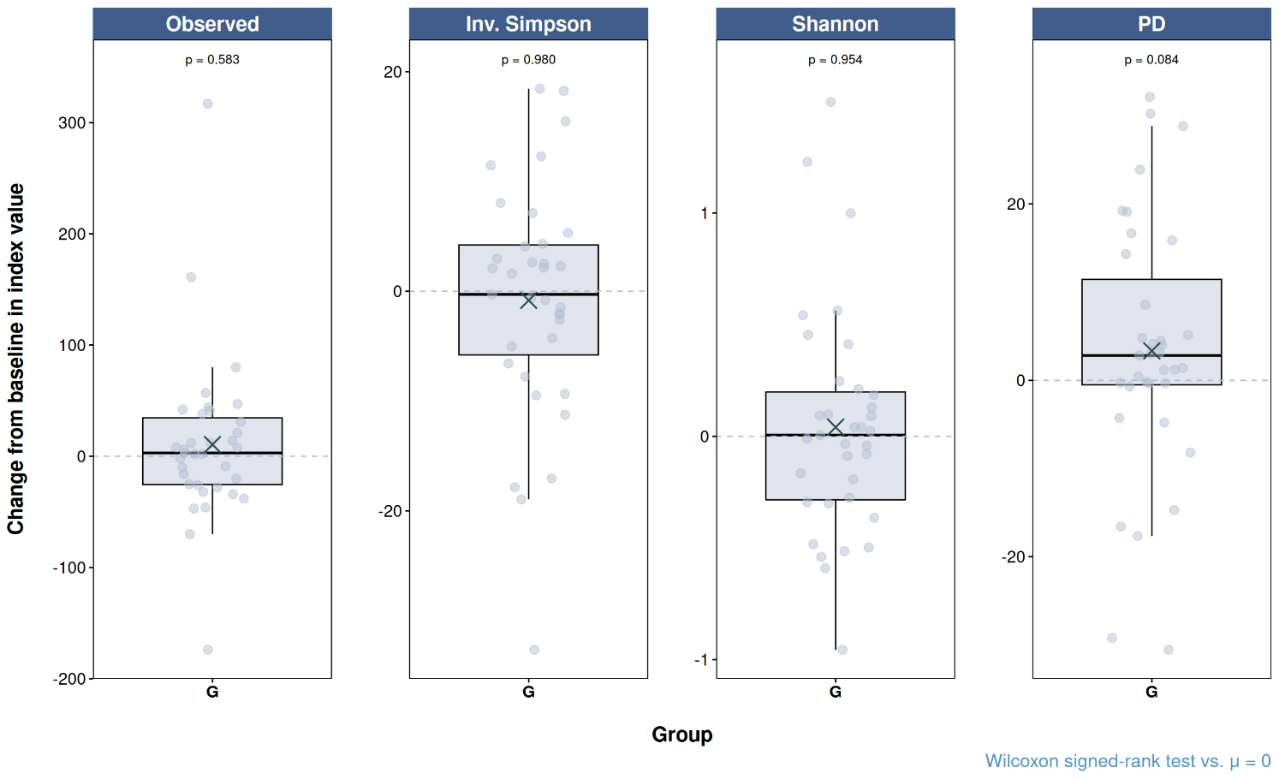


**Supplementary Figure 1.** Evolution of α-diversity indices of fecal microbiota in dogs. Wilcoxon signed-rank test. Change from baseline (D0) to end of supplementation (D28).


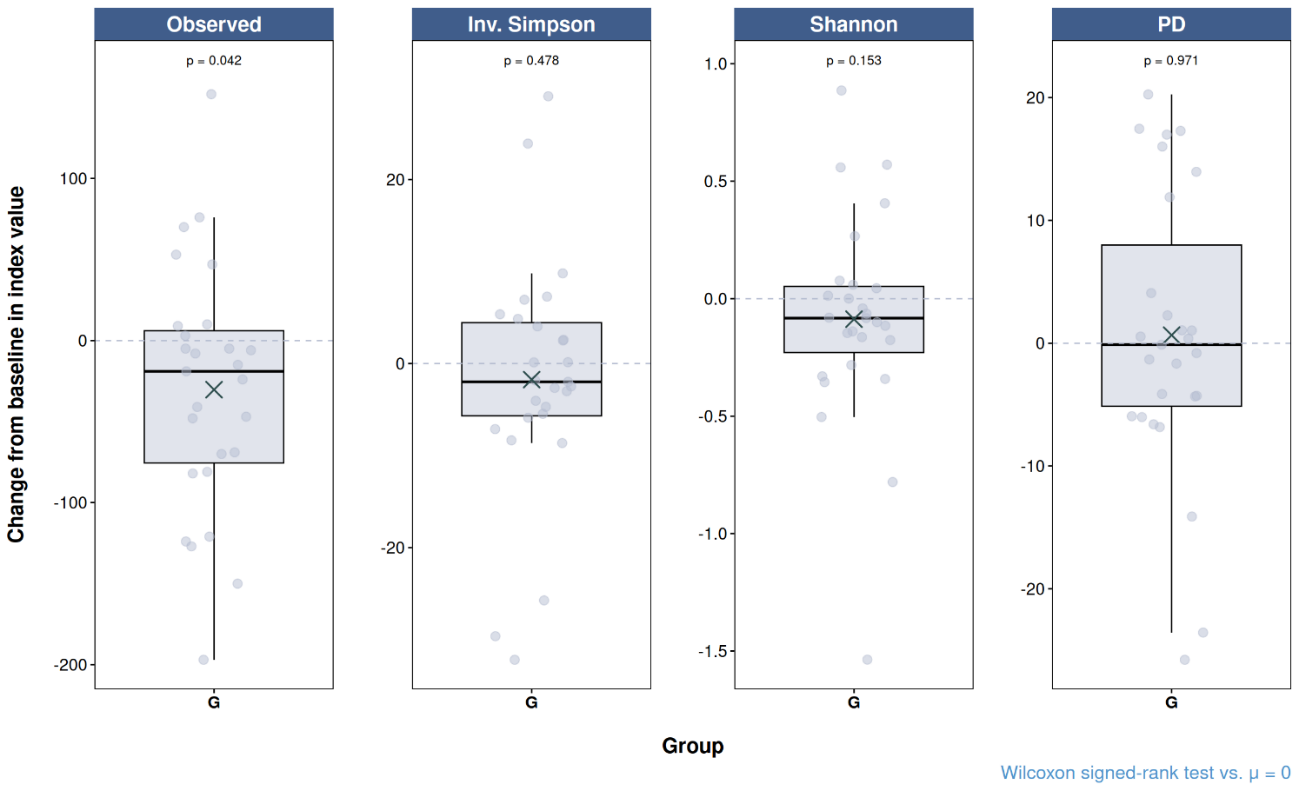


**Supplementary Figure 2.** Evolution of α-diversity indices of fecal microbiota in cats. Wilcoxon signed-rank test. Change from baseline (D0) to end of supplementation (D28).

## Supplementary Table

| **Species** | **Distance metric** | **P-value** |
| --- | --- | --- |
| Dog | Bray-Curtis | 0.1094 |
|  | Jaccard | 0.0796 |
|  | wUniFrac | 0.2336 |
|  | Bray-Curtis | 0.2204 |
|  | Jaccard | 0.0221* |
|  | wUniFrac | 0.5939 |

**Supplementary Table 1.** Within-group beta-diversity comparison. Results from stratified PERMANOVA (n=9999 permutations). ***P<0.001; **P<0.01; *P<0.05.

# Bibliography

1. Klindworth A, Pruesse E, Schweer T, Peplies J, Quast C, Horn M, et al. Evaluation of general 16S ribosomal RNA gene PCR primers for classical and next-generation sequencing-based diversity studies. Nucleic Acids Res. 7 janv 2013;41(1):e1.

2. Ansorge R, Birolo G, James SA, Telatin A. Dadaist2: A Toolkit to Automate and Simplify Statistical Analysis and Plotting of Metabarcoding Experiments. Int J Mol Sci. 18 mai 2021;22(10):5309.

3. Magurran AE. Measuring Biological Diversity. Blackwell Publ Oxf. 2004;

4. Faith DP. Conservation evaluation and phylogenetic diversity. Biol Conserv. 1992;61(1):1‑10.

5. Legendre P, Legendre L. Numerical Ecology [Internet]. 2012 [cité 27 nov 2025]. Disponible sur: https://shop.elsevier.com/books/numerical-ecology/legendre/978-0-444-53868-0

6. Lozupone CA, Hamady M, Kelley ST, Knight R. Quantitative and Qualitative β Diversity Measures Lead to Different Insights into Factors That Structure Microbial Communities. Appl Environ Microbiol. mars 2007;73(5):1576‑85.

7. Wilson N, Zhao N, Zhan X, Koh H, Fu W, Chen J, et al. MiRKAT: kernel machine regression-based global association tests for the microbiome. Bioinformatics. 12 juill 2021;37(11):1595‑7.

8. Fernandes AD, Reid JN, Macklaim JM, McMurrough TA, Edgell DR, Gloor GB. Unifying the analysis of high-throughput sequencing datasets: characterizing RNA-seq, 16S rRNA gene sequencing and selective growth experiments by compositional data analysis. Microbiome. 5 mai 2014;2(1):15.
